# A toolbox for epitope-tagging and genome-wide location analysis in *Candida albicans*

**DOI:** 10.1186/1471-2164-9-578

**Published:** 2008-12-02

**Authors:** Hugo Lavoie, Adnane Sellam, Christopher Askew, André Nantel, Malcolm Whiteway

**Affiliations:** 1 Biotechnology Research Institute, National Research Council, Montreal, Quebec, H4P 2R2, Canada; 2 Department of Biology, McGill University, Montreal, Quebec, H3A 1B1, Canada; 3 Department of Anatomy and Cell Biology, McGill University, Montreal, Quebec, H3A 1B1, Canada

## Abstract

**Background:**

*Candida albicans *is a diploid pathogenic fungus not yet amenable to routine genetic investigations. Understanding aspects of the regulation of its biological functions and the assembly of its protein complexes would lead to further insight into the biology of this common disease-causing microbial agent.

**Results:**

We have developed a toolbox allowing *in vivo *protein tagging by PCR-mediated homologous recombination with TAP, HA and MYC tags. The transformation cassettes were designed to accommodate a common set of integration primers. The tagged proteins can be used to perform tandem affinity purification (TAP) or chromatin immunoprecipitation coupled with microarray analysis (ChIP-CHIP). Tandem affinity purification of *C. albicans *Nop1 revealed the high conservation of the small processome composition in yeasts. Data obtained with *in vivo *TAP-tagged Tbf1, Cbf1 and Mcm1 recapitulates previously published genome-wide location profiling by ChIP-CHIP. We also designed a new reporter system for *in vivo *analysis of transcriptional activity of gene *loci *in *C. albicans*.

**Conclusion:**

This toolbox provides a basic setup to perform purification of protein complexes and increase the number of annotated transcriptional regulators and genetic circuits in *C. albicans*.

## Background

*Candida albicans *is an important human fungal pathogen because of its clinical significance as well as its use as an experimental model for scientific investigation [1]. This opportunistic pathogen is a natural component of the human skin, gastrointestinal and genitourinary flora, but it can sporadically cause a variety of infections. Although many *Candida *infections are not life-threatening (oral thrush and vaginal candidiasis, for example), immunosuppressed patients can be subjected to potentially lethal systemic infections, and therefore *Candida *infections are a major public health concern [2,3]. *C. albicans *can also colonize various biomaterials, and readily forms dense, complex biofilms that are resistant to most antifungal agents. Because of the challenges of drug resistance [4] and the eukaryotic nature of *C. albicans *that makes it similar to its human host, extensive efforts are underway to identify new drug targets for therapeutic intervention; many of these take advantage of tools from the genomic era [5-7]. Because of some of its unique biological features, *C. albicans *is also becoming attractive to studies of more fundamental aspects of genome maintenance, regulatory biology and morphogenesis [8-10].

The diploid nature and the absence of a complete sexual cycle in *C. albicans *reduces the ease with which genetic manipulations can be achieved [11]. Therefore, biochemical, cell biological and genomic analyses of gene products provide alternate strategies to improve our understanding of this pathogen. In particular, obtaining a better insight into how specific protein complexes assemble in *C. albicans *would help define targets for drug development. For example, the large-scale definition of protein complexes by tandem affinity purification (TAP), as it was previously done in *S. cerevisiae *[12,13], should reveal the architecture of biochemical networks and protein machines specific to *C. albicans*. This approach would also give insight into the evolution of protein complexes in ascomycetes. As well, the availability of tools allowing comprehensive functional analysis of transcription factors (TFs) on a genome-wide scale could enhance cellular studies. Genome-wide location analysis, or ChIP-CHIP analysis (chromatin immunoprecipitation (ChIP) followed by DNA microarrays (CHIP)) allows the identification of direct targets of a defined TF on a genomic scale [14]. This approach has been comprehensively applied to the model budding yeast *S. cerevisiae *in order to map its transcriptional regulatory network [15,16]. While first developed in *S. cerevisiae*, ChIP-CHIP has since been applied to other organisms including *C. albicans *[17,18]. In addition, *C. albicans *has recently been established as an interesting model organism for the study of the evolution of transcriptional regulatory networks. In fact, it appears that *C. albicans *differs significantly in its mode of gene regulation from the well-characterized *S. cerevisiae *transcriptional circuits. It has recently served as a comparative system for studying the evolution of the mating type control circuit and the evolution of ribosomal protein (RP) regulation [19,20]. Furthermore, changes occuring in the activity of TFs seem to account for part of acquired drug resistance in *C. albicans *[21,22]. Moreover, several TFs play a critical role in the *C. albicans *morphological transitions and in biofilm formation [10,23]. Therefore, a detailed understanding of the transcriptional regulation mechanisms in *C. albicans *would be valuable for basic research purposes as well as for improving our understanding of drug resistance and for aiding in the development of new antifungals.

Here we report the construction of a new set of PCR-based epitope-tagging vectors for *C. albicans *that is successfully applied to perform a biochemical characterization of the small processome subunit via Nop1 tandem affinity purification as well as genome-wide location analysis of the model TFs Tbf1, Cbf1 and Mcm1.

## Results and discussion

### A set of PCR cassettes for protein tagging

**Figure 1:**
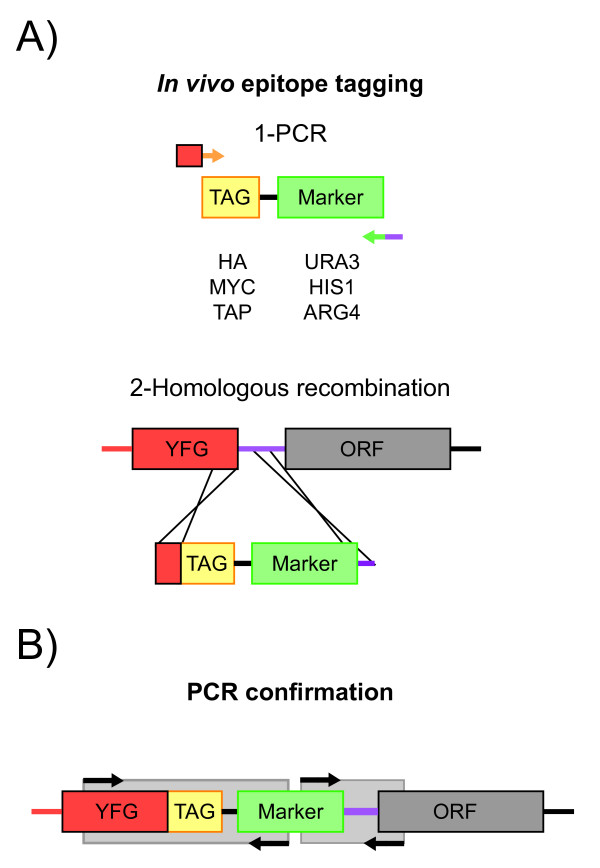
**Plasmid constructs for *in vivo *protein tagging in *C. albicans***. A) Two-step insertion of tags by 1- PCR and 2- homologous recombination. B) PCR confirmation of tagged constructs. YFG stands for Your Favorite Gene.

The choice of selectable marker genes in our cassettes, *URA3, HIS1 *and *ARG4 *was guided by the availability of the *C. albicans *strains BWP17 and SN76 that have the auxotrophic mutations (*ura3*/*ura3*; *his1*/*his1*; *arg4*/*arg4*)[24,25]. Our cassettes permit the use of a single 120-bp primer pair (20 bp of vector sequences and 100 bp from the gene to be tagged) to tag genes with three epitopes (Fig. 1A). In addition, our PCR strategy is compatible with the previously published pFA-XFP-tagging system [26]. With this vector set, the tagged protein is expressed under the control of its own regulatory sequences and chromatin environment and thus at its normal physiological levels, thereby maximizing the biological relevance of biochemical analyses.

**Figure 2:**
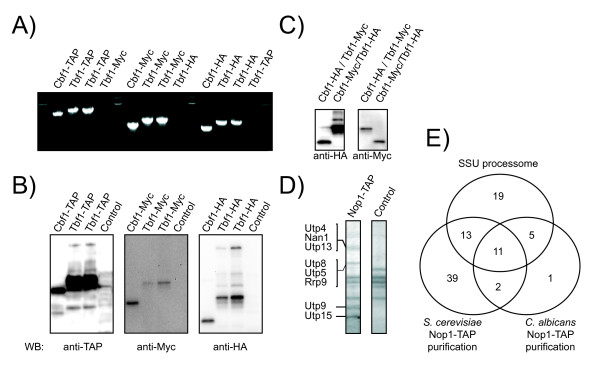
**Our new PCR tagging cassettes allow the rapid biochemical analysis of tagged proteins**. PCR (A) and Western blot (B) confirmation of TAP, Myc and HA-tagged transcription factors Cbf1 and Tbf1. C) Western blot validation of doubly tagged strains Cbf1-HA/Tbf1-Myc and Cbf1-Myc/Tbf1-HA. D) SYPRO Ruby stained SDS-PAGE gel following tandem affinity purification of Nop1-TAP. Proteins identified in each band by in-gel digestion followed by mass spectrometry are identified on the left. E) Venn diagram comparison of Nop1-TAP hits in *C. albicans *and *S. cerevisiae*.

The HA and MYC epitope tags have been used to tag proteins in various organisms and for various applications [27,28]. They represent highly immunogenic peptides with little biological activity that can then be used in immunoprecipitation/co-immunoprecipitation experiments to validate protein complex formation. The TAP tag was more recently developed in order to obtain high purified protein samples for mass spectrometry analysis of protein complexes [29]. Tagged strains were first validated by PCR and subsequently by western blotting (Fig. 2A and 2B), and we confirmed that the tagged constructs are easily immunoprecipitated (data not shown). Doubly tagged strains can also be obtained with combinations of selective markers offered by the BWP17 and the SN76 strain backgrounds. We produced Tbf1-HA/Cbf1-Myc and reciprocal tagged stains (Fig. 2C).

### Tandem affinity purification of Nop1

Since its introduction in 1999, the TAP-tag has been used in various organisms to perform protein-complex purifications [29]. In *S. cerevisiae*, it has been used to assess protein-protein interactions on a large scale [12,30]. We performed a standard TAP procedure using the TAP-tagged Nop1, a component of the small processome subunit in *S. cerevisiae *[13]. Mass spectrometric analysis of in-solution digested TCA-precipitated proteins after the TAP procedure identified 18 *C. albicans *proteins and in-gel digestion of four SYPRO Ruby stained SDS-PAGE bands led to the identification of an additional protein (Fig. 2D and Table 1). In total, 19 *C. albicans *proteins were identified, 16 of which are *bona fide *components of the *S. cerevisiae *small sub-unit processome (SSU processome) (Table 1 and Fig. 2E) [13]. In addition, three ribosomal proteins were co-purified with Nop1; ribosomal proteins are common false positives in protein complex purification procedures (Table 1)[31]. We compared the coverage generated through our approach to that of the orthologous Nop1-TAP purification in *S. cerevisiae*. The *C. albicans *Nop1-TAP bait retrieved 16 proteins (33%) of the annotated SSU processome subunits while its *S. cerevisiae *ortholog retrieved 24 (50%) , so the coverage of the *S. cerevisiae *set was higher. However, the proportion of core components of the SSU processome retrieved in *C. albicans *(84%) surpasses the proportion retrieved in *S. cerevisiae *by all published affinity-capture followed by mass spectrometry studies (37%)[32]. It is likely that the stringency of our protocol accounts for the lower coverage and higher specificity of the set of hits retrieved; Modifications of the purification conditions could allow for the desired specificity. Overall, it is apparent that a basic TAP protocol is amenable to *C. albicans *proteins. This approach will be of great utility in the analysis of function of currently poorly defined proteins and in the study of the evolution of protein complexes in yeasts.

**Table 1:** Mass spectrometry results after Nop1-TAP tandem affinity purification

**Systematic name**	**Common name**	**Protein Score**	Number of peptides*	**Mass (kDa)**	**Cellular component**	**Biological process**	**Molecular function**
orf19.7569	Sik1	504.3	8	58	small-subunit processome	rRNA modification	snoRNA binding

orf19.1566	Utp21	337.4	7	104	Small-subunit processome	rRNA modification	Unknown

orf19.1199	Nop5	327.5	6	57	Small-subunit processome	rRNA modification	snoRNA binding

orf19.7154	Utp18	208.3	4	62	Small-subunit processome	rRNA modification	Unknown

orf19.1633	Utp4	206.5	4	85	Small-subunit processome	rRNA modification	snoRNA binding

orf19.3138	Nop1*	186.5	3	33	Small-subunit processome	rRNA modification	snoRNA binding

orf19.7599	Utp5	174.3	4	71	Small-subunit processome	rRNA modification	Unknown

orf19.3609	Utp15	172.8	2	47	Small-subunit processome	rRNA modification	snoRNA binding

orf19.6710	Utp9	149.8	1	50	Small-subunit processome	rRNA modification	snoRNA binding

orf19.1601	Rpl3	121.4	2	44	Cytosolic large ribosomal subunit	translation	structural constituent of ribosome

orf19.3430	Bud21	100.9	2	25	Small-subunit processome	rRNA modification	snoRNA binding

orf19.3276	Pwp2	99.7	2	104	Small-subunit processome	rRNA modification	snoRNA binding

orf19.2688	Nan1	88.7	2	94	Small-subunit processome	rRNA modification	snoRNA binding

orf19.5436	Utp8	88.5	1	81	Small-subunit processome	rRNA modification	snoRNA binding

orf19.5106	Dip2	77.8	2	110	small-subunit processome	rRNA modification	snoRNA binding

orf19.5225.2	Rpl27	59.7	1	16	cytosolic large ribosomal subunit	translation	structural constituent of ribosome

orf19.2830	Rrp9	58.8	1	62	small-subunit processome	rRNA modification	snoRNA binding

orf19.6085	Rpl16	56.9	1	20	cytosolic large ribosomal subunit	translation	structural constituent of ribosome

orf19.4268	Utp13**	NA	NA	90	small-subunit processome	rRNA modification	snoRNA binding

* Nop1 was the bait in this tandem affinity purification experiment.

** Utp13 was identified by in-gel but not in-solution digestion.

### ChIP-CHIP analysis of *in vivo *tagged transcription factors

**Figure 3:**
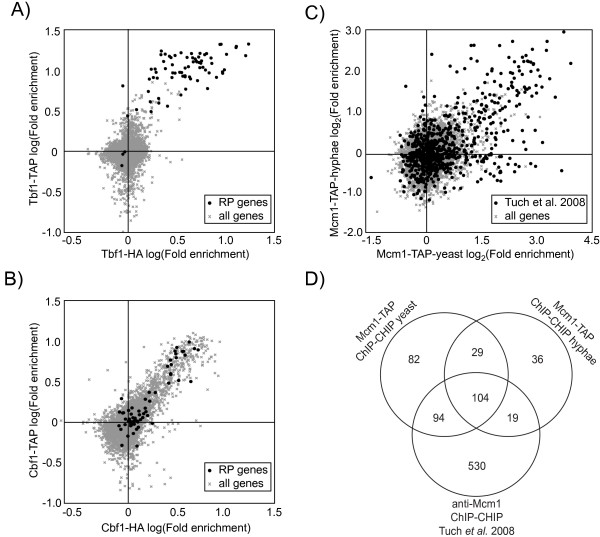
**Validation of the Cbf1, Tbf1 and Mcm1 TAP-tagged TFs by ChIP-CHIP analysis**. Tbf1-HA and Cbf1-HA ChIP-CHIP [20] overlaps Tbf1-TAP (A) and Cbf1-TAP (B) ChIP-CHIP performed with our *in vivo *tagged constructs. C) Comparison of Mcm1-TAP ChIP-CHIP conducted before and after the yeast to hyphal transition with the Mcm1 targets derived from Tuch et al. (2008) experiments highlighted. D) Comparison of the target genes of Mcm1-TAP in yeast and hyphal states and their overlap with Tuch *et al*. (2008) gene list.

One potential application of TAP-tagged TFs consists of their use in ChIP-CHIP to map their DNA-binding targets *in vivo*. ChIP-CHIP was therefore performed for three TAP-tagged TFs; Tbf1, Cbf1 and Mcm1. For Tbf1 and Cbf1, the results obtained were compared to previously published data using ectopically expressed Tbf1-HA and Cbf1-HA constructs [20]. The location profiles of TAP-tagged TFs were essentially identical to the previous results and confirmed that the RP regulon is dominated by the Myb transcription factor Tbf1 working in conjunction with Cbf1 (Fig. 3A and 3B) [20].

Genome-wide location of the *S. cerevisae *MADS box cell cycle regulator ortholog Mcm1 was also investigated in *C. albicans *cells. *S. cerevisiae *Mcm1 plays a critical role in the regulation of cell cycle transitions and, more specifically, of the pre-replication complex (Pre-RC). The Mcm1 paralog Arg80 arose after the whole-genome duplication of the yeast lineage and is involved in the regulation of arginine metabolism genes [8]. The overlap of our Mcm1 genome-wide binding data in yeast state cells with a previously published Mcm1 ChIP-CHIP done on tiling microarrays is highly significant (pvalue = 2.7E-11) with 198 genes common between studies [8] (Fig. 3D). We confirm that *C. albicans *Mcm1 occupies the roles of *S. cerevisiae *Mcm1 and Arg80 since genes from the arginine biosynthesis pathway such as *ARG3/4/5–6/8*, *CAR1 *and the zinc transcription factor *ARG81 *are enriched in our set (pvalue = 5.52E-07; Table 2). Genes involved in the biosynthesis of the other basic amino acid, lysine (*LYS1/9/21/22 *and 144), and also catabolic proline processing (*PUT1 *and *2*) are also enriched in ours and Tuch *et al*. (2008) gene lists (Table 2). In addition, we confirm that Mcm1 binds strongly to its own promoter (Table 2). We finally confirm a previous study revealing that *C. albicans *Mcm1 regulates *MDR1 *expression and thereby, drug resistance [8,33]. Indeed, a significant enrichment (7.49 fold) of Mcm1 binding to the promoter of the drug efflux facilitator gene *MDR1 *was observed in both yeast and hyphal states.

**Table 2:** Mcm1-TAP targets in ChIP-CHIP analysis

**Systematic name**	**Common name**	**Function**	**Fold enrichment**
**Arginine metabolism**			

*orf19.5610*	ARG3*	Ornithine carbamoyl-transferase	2.20

*orf19.4788*	*ARG5,6*	Acetylglutamate kinase	3.24

*orf19.3770*	ARG8*	Acetylornithine aminotransferase	5.18

*orf19.2077*	ARG81*	Zinc-finger TF	3.08

*orf19.3934*	*CAR1*	Arginase	3.82

*orf19.3221*	CPA2*	Carbamoyl phosphate synthetase	2.58

**Lysine metabolism**			

*orf19.1789.1*	LYS1*	Saccharopine dehydrogenase	5.00

*orf19.7448*	LYS9*	Saccharopine dehydrogenase	2.27

*orf19.772*	*LYS21*	Homocitrate synthase	2.58

*orf19.4506*	*LYS22*	Homocitrate synthase	2.57

*orf19.5380*	LYS144*	Zinc cluster TF	2.78

**Proline catabolism**			

*orf19.4274*	PUT1*	Proline dehydrogenase	5.01

*orf19.3974*	PUT2*	1-pyrroline-5-carboxylate dehydrogenase	2.78

**Drug resistance**			

*orf19.5604*	*MDR1*	ABC transporter	

**Mcm complex**			

*orf19.7025*	MCM1*	MADS box TF	5.59

*orf19.4354*	MCM2*	ATP-dependent DNA helicase	3.65

*orf19.1901*	*MCM3*	ATP-dependent DNA helicase	4.1

*orf19.3761*	*MCM4*	ATP-dependent DNA helicase	3.28

*orf19.5487*	*MCM5*	ATP-dependent DNA helicase	2.95

*orf19.201*	MCM7*	ATP-dependent DNA helicase	3.87

**Mcm complex loading**			

*orf19.5242*	CDC6*	ATPase helicase clamp loader	1.79

**Origin recognition complex (ORC)**			

*orf19.3000*	*ORC1*	ATPase for DNA binding	1.67

*orf19.6942*	*ORC3*	ATPase	1.81

* Genes in common with ChIP-CHIP data of Tuch et al., (2008) using tiling array.

However, our data diverges from the other Mcm1 ChIP-CHIP publications at some points. First, our results show Mcm1 binding in the promoters of almost all members of the pre-replication complex (Pre-RC) (Mcm helicases genes *MCM2/3/4/5 *and *7*; pvalue = 1.87E-06; Table 2) while the Tuch *et al*. (2008) data only uncovered two members of this complex (*MCM2 *and *7*). In addition, we detected reproducible Mcm1 binding at two origin-recognition-complex components (*ORC1 and 3*) and the loading factor for the Mcm complex, *CDC6 *(Table 2; see Additional file 1). Second, binding of the promoters of the cyclin genes *CLN3, HGC1 *and *CLB3 *by Mcm1 was not detected in our experimental setup (see Additional file 1 and 2). In total, 530 Mcm1 targets are uniquely found in the Tuch et al. gene list [8](Fig. 3D). These major differences are likely due to variations between the two independently conducted studies. Tuch and collaborators 1) treated their cells with pheromone, 2) used a polyclonal rabbit antiserum raised against an Mcm1 peptide, 3) used signal ratios of cy5 labelled IP versus cy3 labelled whole-cell extract prior to performing the IP and 4) used tiling arrays to determine the promoter targets of Mcm1. Our procedure is somewhat different: we 1) used YPD grown cells, 2) used IgG beads-proteinA interaction to enrich target regions, 3) compared each experimental IP to a mock IP performed in untagged cells and 4) used full genome arrays with smaller coverage (about two probes/intergenic region). All these experimental changes could affect to different degrees the final result of a ChIP-CHIP. First, treating cells with pheromone might dramatically alter Mcm1 binding at some point in the cell cycle and pheromone response elements and deplete it at others (such as the Mcm complex genes). Second, polyclonal anti-Mcm1 antibodies can have cross-specificities that would not be corrected for by using the whole-cell extract as a control instead of a mock IP or an IP performed with a preimmune serum. Third, the different resolutions of the two studies could account for some overlooked targets in our experiments.

We also performed Mcm1-TAP ChIP-CHIP in the yeast to hyphal transition triggered by serum at 37°C. Mcm1 was recruited to a limited set of genes (36) under these conditions (Fig. 3D). Noticeably, it was enriched in the promoters of *ALS3*, *HWP1 *and *ECE1 *after hyphal induction (see Additional files 1 and 2).

Thus, our genome-wide location data showed a significant consistency with previously published results on the three TFs. This suggests that our chromosomally tagged alleles are functional and that the use of a TAP-IgG pull-down protocol is applicable for ChIP-CHIP as previously reported in *S. cerevisiae *^26 ^and that this protocol is comparable to a ChIP method based on a anti-HA (for Cbf1 and Tbf1) or rabbit polyclonal (for Mcm1) antibody IP.

### *In vivo *beta-galactosidase reporter assays

**Figure 4:**
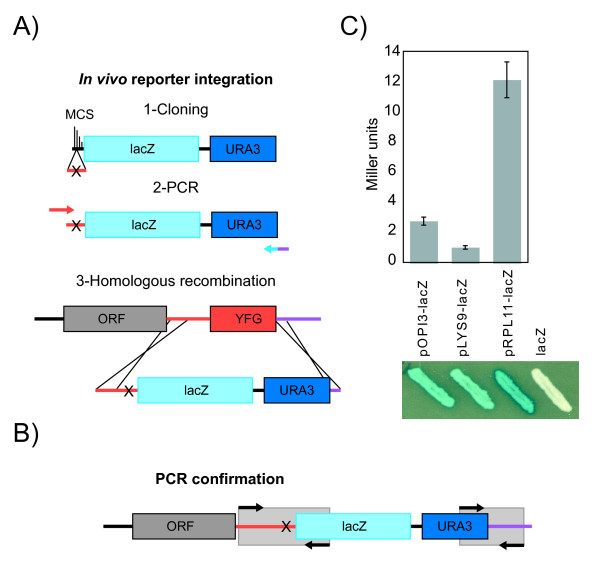
**A new reporter construct allowing*****in vivo *****analysis of gene expression in*****C. albicans***. A) Gene replacement by beta-galactosidase following 1- wild-type or mutant promoter cloning, 2- PCR amplification of the promoter-lacZ fusion and 3- *in vivo *gene replacement by transformation and homologous recombination. B) PCR confirmation of our *in vivo *inserted beta-galactosidase constructs. C) Beta-galactosidase activity of the promoters of the *OPI3*, *LYS9 *and *RPL11 *genes. YFG stands for Your Favorite Gene.

The beta-galactosidase reporter system described here has already been applied to the problem of RP gene regulation in Hogues *et al *(2008). It allows the integration of reporter constructs in the actual chromatin environment of the gene of interest. Here, we describe its usefulness in the quantitative study of active gene loci. We produced p*LYS9*-lacZ and p*OPI3*-lacZ strains and beta-galactosidase reporter assays were performed with YPD grown cells. Our results show variable levels of reporter activity across these three promoters (Fig. 4). The multiple cloning site of our pFA-lacZ-*URA3 *construct allows the easy creation of various *cis*-regulatory mutants as examplified by the the p*RPL11 *promoter (p*RPL11*) [20].

## Conclusion

Here, we have presented a new set of tools for functional characterization of *C. albicans *gene products by biochemical methods. We believe that the availability of such tools will greatly help future understanding of the biology of this important human pathogen.

## Methods

### Construction of chromosomal tagging PCR cassettes

We first adapted the *S. cerevisiae *PCR TAP-tagging vector pEB1340 [34] for its use in *C. albicans *by substituting the *S. cerevisiae HIS3 *marker with the *C. albicans URA3, HIS1 *and *ARG4 *genes from the previously published pFA-GFP plasmid series [26] (Fig. 1A). Subcloning of the *C. albicans *auxotrophic markers was done by ligation of AscI-PmeI fragments in AscI-PmeI digested pEB1340. We then derived triple HA or MYC epitope-tagging vectors in the same pFA plasmid backbones by cloning oligonucleotides encoding the HA or MYC epitope tags and containing XmaI and AscI sites (Table 3) between the XmaI and AscI sites of the pEB1340 plasmid. Auxotrophic markers *URA3, HIS1 *and *ARG4 *from plasmids pFA-XFP [26] were then subcloned into the HA and Myc constructs between AscI and PmeI.

**Table 3:** Primers used for in this study

Name	Sequence
HA_F	CCGGGTACCCATACGATGTTCCTGACTATGCGGGCTATCCCTATGACGTCCCGGACTATGCAGGATATCCATATGACGTTCCAGATTACGCTTAGGG

HA_R	CGCGCCCTAAGCGTAATCTGGAACGTCATATGGATATCCTGCATAGTCCGGGACGTCATAGGGATAGCCCGCATAGTCAGGAACATCGTATGGGTAC

5MYC_F	CCGGGGAACAGAAGCTTATATCCGAAGAAGACCTCGGAGAGCAAAAGCTCATTTCAGAAGAGGATCTAGGCGAACAGAAACTAATCTCGGAGGAGGACCTCGGTGAACAAAAGCTTATCTCTGAGGAAGATCTTGGCGAGCAGAAGCTCATATCAGAGGAAGACCTAGGG TAG GG

5MYC_R	CGCGCCCTACCCTAGGTCTTCCTCTGATATGAGCTTCTGCTCGCCAAGATCTTCCTCAGAGATAAGCTTTTGTTCACCGAGGTCCTCCTCCGAGATTAGTTTCTGTTCGCCTAGATCCTCTTCTGAAATGAGCTTTTGCTCTCCGAGGTCTTCTTCGGATATAAGCTTCTGTTCC

Nop1-TAP_F	AGAAGAAAGAATTAAACCATTGGAACAATTGACCTTGGAACCTTATGAAAGAGACCATTGTATTGTTGTTGGTAGATACATGAGAAGCGGAATAAAGAAAGGT CGA CGG ATC CCC GGG TT

Nop1-TAP_R	TAGAGTTGATTAGACCTTATTGTTTTATTTTTCATTTTATTATATATGTCGTATCTTACAGTTCTTTAAATACCAGTGTTTCCAAAATTTTCATTCATTCTCG ATG AAT TCG AGC TCG TT

Cbf1-TAP_F	TGAACAAGCTGTTAGTGAATTGAGTGCTTCAAATGAGAAATTGAAACATGAATTAGAATCAGCTTATCGTGAAATCGAACAATTGAAGAGAGGGAAGAAAGGT CGA CGG ATC CCC GGG TT

Cbf1-TAP_R	TAACATAATTTCAAATACCGAGTAGGAATACACAACCCCAACATCTAACCAGCCATACATTTACATATTTATAATTACATATTAAAACATCGTCAAATTAATCG ATG AAT TCG AGC TCG TT

Tbf1-TAP_F	AACAACAAGAGAAAGAACAACCGGATCAGCAACAACCAGATCAACAACACCCAGATCGACAACAACAAGAGCAGATCCAACAACCAGAAAATCTGGATAAAGGT CGA CGG ATC CCC GGG TT

Tbf1-TAP_R	ATCAACTATTGTGATCCTGCTTAAGTTAGCTTGAACAATTATTCAAATCAATTTACACCTTAAAGATAGATTAATTAACAATACAAATATAATGCTACATGTCG ATG AAT TCG AGC TCG TT

Mcm1-TAP_F	ACATCAACCTGGTATTCCATTACAAGGTGGTTATAGTGATCAATACCTGTATTTTGGTAATATTCAAAATAACAACATACCTAATCAACAGCAATATCAAGGTCGACGGATCCCCGGGTT

Mcm1-TAP_R	ATTATTCACCTAAATCCCCTGACCTCTGGCCAAACACTTTCTTTGTAGATGGGAGGGGAGCGGGGGGAGGAAATGAAAAACCATTGGCAACGAGAAAAAGATCGATGAATTCGAGCTCGTT

Nop1_confirm_F	AATCATAGATGATGCTAGACATCC

Nop1_confirm_R	TGTTGGGTCTAAAGGTCAAAGTGC

Cbf1_confirm_F	AAGAGAATCCATAAATACTGG

Cbf1_confirm_R	CTTTGATACCCCTAATGTTTC

Tbf1_confirm_F	AAGCGGTTTGGAAATTCCTAG

Tbf1_confirm_R	TTGCATGTTAAAATTCGTCTC

Mcm1_confirm_F	TGACGATGGTACTTCTCAAGGT

Mcm1_confirm_R	AATTGTTTCAACATTTTGGTTTTT

TAP-HA_F	GGT CGA CGG ATC CCC GGG TTA TAC CCA TAC GAT GTT CCT GAC

TAP-MYC_F	GGT CGA CGG ATC CCC GGG TTA GAA CAG AAG CTT ATA TCC GAA

TAP-HA-MYC_R	TCG ATG AAT TCG AGC TCG TT

The beta-galactosidase reporter was constructed by subcloning a PstI-MluI fragment corresponding to the *Streptococcus thermophilus *lacZ ORF from plasmid placpoly [35] between the PstI and AscI sites of plasmid pFA-XFP-URA3 [26].

PCR reactions were performed in 50-μl volumes containing 1 ng of plasmid template with the Expand Long Template polymerase following manufacturer's instructions (Roche, Germany). PCR parameters were 1 cycle at 94°C, 5 min followed by 35 cycles at 94°C, 30 sec; 58°C, 1 min; 68°C, 3 min.

### Construction of *C. albicans *epitope-tagged strains

Cell growth, transformation, and DNA preparation were carried out using standard procedures [26,36]. Transformants were selected on either of -Ura, -His or -Arg selective plates. Correct integration was verified by PCR, sequencing and finally Western blotting (Fig. 1A). Rate of correct integration was comparable to a previous study using similar condiitons and was in the range of 40–80% depending on the gene locus considered [26]. We used this strategy to C-terminally fuse Tbf1, Cbf1 and Mcm1 with a TAP tag and to introduce an HA or a MYC tag to the C-terminus of Tbf1 and Cbf1.

### Western blotting

Whole cell extracts were obtained by boiling cells at 2 ODs in loading buffer with 100 mM DTT for 10 minutes. Proteins were then separated on a 10% SDS-PAGE gel and transferred to a PVDF membrane (Millipore). Antibodies were prepared in TBS-0.05% Tween20 5% skim milk powder. A rabbit polyclonal antibody directed against the TAP-tag (Open Biosystems) was used at 0.5 μg/ml while monoclonal antibodies anti-HA (12CA5) and anti-Myc (9E10) were used at 5 μg/ml. HRP-conjugated goat anti-rabbit and anti-mouse secondary antibodies (Santa Cruz) were used at 0.04 μg/ml. The HRP signal was revealed with Immobilon™ HRP substrate (Millipore).

### TAP purifications and mass spectrometry

Tandem affinity purifications were performed as described  and then precipitated with Trichloroacetic acid (TCA). For mass spectrometry analysis of the TAP purified proteins, the digestion was performed using Trypsin (Promega) in 50 mM ammonium bicarbonate for 4 hours at 37°C and dried down. One quarter of the TCA precipitate was loaded on a 10% SDS-PAGE gel. The gel was stained with SYPRO Ruby according to manufacturer's instructions (Invitrogen). Excised protein bands were processed as described [37]. Samples were resolubilized in 5% acetonitrile 0.2% formic acid and analyzed on a Eksigent nanoLC system coupled to a Thermo LTQ-Orbitrap MS instrument with a home-made C18 pre-column (5 mm × 300 um) and an analytical column (10 cm × mm × 300 m i.d. Jupiter 3 m C18). Sample injection was 10 ul. The digest was first loaded on the pre-column at a flow rate of 4 ul/min and subsequently eluted onto the analytical column using a gradient from 10% to 60% aqueous acetonitrile (0.2% formic acid) over 56 min at 600 nl/min. Database searches were performed against a non-redundant fungal database using Mascot version 2.1 (Matrix Science).

### ChIP-CHIP analysis of Tbf1, Cbf1 and Mcm1by TAP-IgG pull-down in *C. albicans*

Chromatin immunoprecipitation (ChIP) experiments were performed with chromosomally tagged Tbf1-TAP, Cbf1-TAP and Mcm1-TAP as described [20]. Cells were grown to an optical density at 600 nm of 0.6 in 50 ml of YPD or YPD with 10% FBS for Mcm1-TAP in hyphal state. We followed the ChIP protocol available at  with the following exceptions: chromatin was sonicated to an average 300 bp, and 700 μl of whole-cell extract (WCE) were incubated with IgG-Sepharose beads (GE Healthcare). Tagged ChIPs were labeled with Cy5 dye and untagged (mock) ChIPs were labeled with Cy3 dye and were then co-hybridized to our full-genome arrays.

### *Candida albicans *full-genome arrays, hybridization, scanning and normalization

Our *C. albicans *full-genome microarrays contain single spots of 5,423 intergenic 70-mer oligonucleotide probes combined with 6,394 intragenic 70-mer oligonucleotide probes already in use in our *C. albicans *ORF microarray [20,38]. We designed the 5,423 probes that correspond to the promoter regions of most of the genes in the *C. albicans *Genome Assembly 21 by using the same algorithm that was successfully applied to the development of our *C. albicans *ORF oligonucleotide arrays with added weight provided for regions of high homology among *Candida *species. Our lab has developed full-genome (ORF and intergenic) arrays for use in location profiling experiments and the DNA microarrays were processed and analysed as previously described [20].

### Nucleotide sequence accession numbers

The sequences of plasmids pFA-TAP-*URA3*, pFA-TAP-*HIS1*, pFA-TAP-*ARG4*, pFA-HA-*URA3*, pFA-HA-*HIS1*, pFA-HA-*ARG4*, pFA-MYC-*URA3*, pFA-MYC-*HIS1*, pFA-MYC-*ARG4 *and pFA-LacZ-*URA3 *have been submitted to GenBank and have been assigned the following accession numbers: FJ160456, FJ160457, FJ160458, FJ160464, FJ160462, FJ160463, FJ160460, FJ160461, FJ160459 and FJ160455, respectively.

## Authors' contributions

HL carried out cloning procedures, HA, MYC and TAP tagging of Cbf1 and Tbf1 in *C. albicans*, immunoblots, tagging and affinity purification of NOP1-TAP, ChIP-CHIP of Cbf1-TAP and Tbf1-TAP as well as *lacZ *reporter assays. AS and CA constructed the Mcm1-TAP *C. albicans *strain and performed ChIP-CHIP of Mcm1-TAP in both yeast and hyphal states. AN and MW oversaw the work. The first draft of the article was written by HL and AS. All authors read and approved the final manuscript.

## Supplementary Material

Additional file 1 **Promoter targets of Mcm1 in our and Tuch*****et al***. (2008) data. The data provided represent gene promoter bound by Mcm1 in both yeast and hyphae conditions. Tuch *et al*. (2008) Mcm1 binding dataset was also included. Click here for file

Additional file 2 **Mcm1-TAP ChIP-CHIP fold changes in yeast and hyphal states**. This file contains the complete data of Mcm1 ChIP-CHIP experiment performed in this study. Click here for file
